# Experience of Youths and Older People With Virtual Reality Games for Cognitive Assessment: Inductive Thematic Analysis and Insights for Key Stakeholders

**DOI:** 10.2196/59197

**Published:** 2024-06-28

**Authors:** Yesoda Bhargava, Veeky Baths

**Affiliations:** 1 Cognitive Neuroscience Lab Department of Biological Sciences Birla Institute of Technology and Science, Pilani - K. K. Birla Campus Goa India

**Keywords:** virtual reality, cognitive assessment games, inductive thematic analysis, youth, older adult, cognitive, cognitive assessment, virtual reality games, game, games, thematic analysis, neurological, utility, cognitive assessment tools, game based, cognitive games

## Abstract

**Background:**

Virtual reality (VR)–based goal-oriented games for cognitive assessment are rapidly emerging and progressively being used in neuropsychological settings. These games have been validated quantitatively, but minimal qualitative insights from users currently exist. Such insights on user experience are essential to answering critical questions linked to the games’ large-scale usability, adoption in hospital settings, and game design refinement. Current qualitative studies on these games have used general questionnaires or web-based reviews to answer these questions, but direct observation from primary settings is missing. We believe that direct observation of participants playing these games and subsequent interaction with them is critical to developing a more objective, clear, and unbiased view of the games’ efficacy, usability, and acceptability.

**Objective:**

In this study, we aimed to extract constructive and relevant insights directly from the participants who played VR-based goal-oriented games. We used these insights to answer vital questions linked to the practical utility of VR-based cognitive assessment. On the basis of these results, we also aimed to provide actionable insights to key stakeholders in the field, such as researchers, game developers, business personnel, and neuropsychology and allied professionals.

**Methods:**

Interview data from 82 younger (aged 18-28 years) and 42 older adult (aged >60 years) participants were used. The interview data were obtained from the 2 pilot studies we conducted on VR games for cognitive assessment. Inductive thematic analysis was conducted on the interview data, and later, the findings were carefully interpreted to develop implications for the key stakeholders.

**Results:**

We identified 5 themes: ergonomic issues, learning and training, postgame effects, game feedback, and system purpose. Regarding hardware, headset weight, adjustment straps, and controllers need to be improved to promote easy use of the device. Regarding software, graphics quality, immersion experience, and game mechanics are the primary deciding factors for a positive user experience. The younger group prioritized purpose and utility for long-term use, whereas the older participants cherished the entertainment aspect. Researchers and game developers must conceptualize and develop games that can provide maximum insights into real-world abilities. Manufacturing businesses need to improve the headset and accessories to make them more user-friendly. Finally, neuropsychology and allied practitioners must identify strategies to engage and train the participants to try VR-based cognitive assessment games.

**Conclusions:**

VR-based games for cognitive assessment are promising tools to improve the current practices of neuropsychological evaluations; however, a few changes are required to make the overall user experience enjoyable, purposeful, and sustainable. In addition, all the key stakeholders need to focus on meaning and purpose over the hype of VR and are advised to work in synergy.

## Introduction

### Background

Games for cognitive assessment have become very popular [1-6]. This development is motivated by the urgency for early detection of cognitive deficits such as dementia, which do not have a cure [7]. Moreover, traditional methods for cognitive assessment provide limited assistance for early detection of cognitive decline and fail to instruct about the real-world cognitive abilities of people [8-10]. In this context, goal-oriented games can serve as novel tools to address the gaps associated with traditional neuropsychological assessments [11].

Games for cognitive assessment can be either immersive or nonimmersive. The nonimmersive games are played on mobile-, tablet-, or computer-based platforms, whereas immersive games use virtual reality (VR) devices, usually a head-mounted display (HMD) and handheld controllers. Games in both the immersive and nonimmersive environments are effective for evaluating and assessing specific cognitive abilities such as memory, language, spatial abilities, and executive functioning and assist in stroke [12-15] and traumatic brain injury rehabilitation [16,17].

Of the 2 types of games, immersive games have generated a lot of excitement and enthusiasm among researchers, clinical personnel, and commercial enterprises [18]. The VR framework can create interactive and immersive 3D environments, which can simulate the real world. This simulation-based realism of VR environments is conducive to ecologically valid cognitive assessment [19-21]. In fact, VR games can assess the ability to carry out activities of daily living [22], detect visuospatial deficits [23-25], and assess cognition in general [26]. In addition to cognitive assessment, VR games are used for rehabilitation of executive functioning [27,28], memory impairment control [29-31], language improvement [32,33], and motor rehabilitation [34].

Clearly, VR games are useful for cognitive assessment and rehabilitation. Research studies have validated this usefulness through statistical and correlational analyses [22-24,35], and systematic reviews and meta-analyses also confirm the advantages [36-38]. However, quantitative validation is insufficient to deduce the acceptability and adoption of these tools in real-world neurological or medical settings. In fact, according to a qualitative study on 3 focus groups and 60 one-to-one interviews, the functional aspect of technology has little impact on its adoption [39].

Skewed focus on the quantitative validation of VR games exaggerates their functional aspects while completely ignoring the emotional, social, and epistemic aspects that are crucial to their adoption [40,41]. For a fuller validation of these games, it is essential to know users’ attitudes toward them; the complexity they face during use; and the extent of compatibility between the technology and their needs, values, and experiences. These factors can inform the perceived usefulness and ease of use of such VR games, ultimately indicating the likelihood of their adoption in the real world. The US Food and Drug Administration also advocates for qualitative evidence from key stakeholders on VR tools’ relevance and significance [42].

While numerous reviews have established the impact of VR games for measuring and improving cognitive abilities such as executive functioning, spatial reasoning, memory, language, and attention, as well as activities of daily living [43], we still know very little about how users actually feel about these games. Therefore, to obtain perspectives beyond functionality in the context of VR games for cognitive assessment, studies that review and inform user experience and feedback are vital. Existing reviews in this context focus on web-based data [44-46], lack age-specific investigation [44-46], or simply focus on game design elements [47,48]. This lack of focus and direction from the user point of view is detrimental and fruitless to inform the acceptance and adoption of these tools in the real world.

In this context, we undertook this thematic analysis and distilled feedback on VR games for cognitive assessment obtained from 2 cohorts: 93 younger (aged 18-28 years) and 53 older (aged >60 years) participants. Data were obtained from 2 field trials conducted by us. The younger cohort was recruited from the Birla Institute of Technology and Science (BITS) Pilani K. K. Birla Goa Campus, and the older cohort was recruited from the Annasawmy Mudaliar General Hospital, Bangalore, India. All participants were cognitively healthy except for 2 in the older cohort who reported mild cognitive impairment.

Using thematic analysis, we extracted practical, applied, and insightful themes that inform about the strengths, challenges, and limitations of the VR games for cognitive assessment. We discussed the obtained themes and substantiated them through user comments. In a separate section for stakeholders, we discussed the implication of the findings for researchers, game developers, businesses, and medical settings. Our work is relevant to anyone who works at the forefront of using and developing VR games for cognitive assessment and rehabilitation.

In the next section, we present and discuss review studies on immersive and nonimmersive games for cognitive assessment. Later, we briefly describe the methodology used. Finally, the results are presented and discussed, followed by conclusions.

### Literature Review

In this section, we briefly mention studies that quantitatively validate VR-based cognitive assessment games and acknowledge their importance for the translation of these games into the real world. Importantly, we also contrast the significance of qualitative studies on VR-based cognitive assessment games compared to these quantitative validation studies and develop a case for the former’s importance for real-world translation. Finally, we discuss previous qualitative studies on VR games and point out their limitations and contributions and, against this context, justify the significance of our work.

A meta-analysis of 18 studies found that healthy controls scored higher in games for executive functions, visuospatial abilities, and memory as compared to those with cognitive impairment [36]. Accordingly, the authors concluded that VR-based measures for cognitive processes are sensitive in detecting cognitive impairment. A different study established the criterion validity of the VR games for cognitive assessment using 5 factors of personality and convergent validity using scores of computer-based assessments [35], further validating the efficacy of VR-based cognitive assessment. However, the authors also pointed out the need to evaluate these games for their difficulty, adaption, autonomy, and control.

A systematic review of digital games for attention found that quantitative validation of game scores was primarily done against the traditional psychometric counterparts and clinical diagnosis [49] but the enjoyment properties of the games were rarely evaluated. In addition, a meta-analysis and systematic analysis that found VR games to be effective for rehabilitation of older adult patients after stroke remarked the importance of investigating game characteristics that drive positive changes in rehabilitation [37]. In the aforementioned studies, the authors sufficiently proved the validity of the VR games for cognitive assessment but also emphasized the need to examine the qualitative factors to obtain more clarity on the effectiveness and usability of these games. Clearly, there is a consensus among researchers on identifying and examining the factors associated with the wider adoption of these games. In fact, according to the theory of consumer research, knowledge of factors such as emotions, social value, and epistemic value is critical to sense user perception of a product’s usefulness and adoption [40,41]. In this context, we discuss and critically analyze previous studies that inform our qualitative understanding of VR games.

A nonsystematic literature review on VR games for identification and rehabilitation of cognitive disorders reported nausea and disorientation among participants who played the games [50]. Although the authors described the games, the lack of specific insights into the game design and game elements precluded a deeper understanding of the causal factors for nausea and disorientation. Similarly, a narrative review of 29 papers on VR games summarized game designs used in VR game development [47]. However, we do not know how certain game designs are better or more effective than others for the usefulness and ease of use of VR games. On the other hand, a systematic review [48] found that score system and narrative context were the most popular game elements for neuropsychological assessment, training, and rehabilitation, but we do not know how the actual users feel about these popular game elements.

Each of these aforementioned reviews [47,48,50] is limited in its application to the real world as it is based on literature rather than direct user feedback. Although they provide a general understanding of game design, scores, game elements, and their aftereffects, reviews based on direct human experience and feedback are preferable to inform the public-level acceptance and adoption of VR games for cognitive assessment. In fact, direct user opinions and feedback are crucial to ground the VR-based games for cognitive assessment and help separate practical utility from the VR hype [51-54]. To this end, we also discuss some studies that explore user feedback on VR games.

Analysis of 473 VR gamers’ experience found that their use was primarily driven by enjoyment rather than usefulness [44]. Given that the sample comprised dedicated gamers, it is difficult to extrapolate these results to the general populace. Moreover, we do not know which types of games were played by these participants. Thus, even though the sample was large, its utility for reviewing the efficacy of VR games for cognitive assessment is precluded due to lack of information on the types of games played by the participants. Furthermore, the gamers were from different countries, and thus, cultural factors may be responsible for certain VR game preferences [55,56].

In a different study, web-based reviews of VR exergames sold in Steam, VIVEPORT, and Oculus were thematically analyzed, and it was found that realism, intuition, and skill enhancement were associated with positive user engagement, whereas a high number of bugs, poor graphics, and confusing control buttons were associated with user disengagement [45]. Similarly, a study on 1227 experienced VR gamers (6 months of experience) found that display quality, interactivity and service, enjoyment, and perceived control were indirectly linked to acceptance of and continuous intention to play the games [46].

The results of these 2 studies [45,46] provide a broad idea of qualitative aspects of VR games that are favorable and unfavorable for user acceptance, but because these studies are based on web-based reviews [45] and questionnaires [46], their impact is limited and lacks context required for real-world application and influence [52,53,57]. Consequently, it is difficult to discern from the aforementioned studies which features to continue with in VR game development, which to remove, and which to improve for cognitive assessment. To address this gap, it is vital to combine the findings of these reviews with direct feedback from participants in primary settings. Such direct feedback provides clearer information on user attitude toward the games, the challenges they face, and the perceived strengths of the game. Moreover, the opportunity to directly talk to the participants about their experience enriches the evidence base required to objectively establish the acceptance and adoption of VR games for cognitive assessment.

In summary, we observe that the existing evidence on validation of VR games for cognitive assessment is quantitative, lacks discussion and critical analysis of the context and content of the games, and is limited by lack of direct user feedback. Although existing quantitative evidence proves the functionality and potential of these games, it cannot be solely relied upon to indicate the acceptance and adoption of these games in real-world settings such as health centers for older people, old-age homes, neurological clinics, or even at home for individual assessment.

In this context, we present our thematic analysis, which is based on interviews conducted during 2 field trials on VR games for cognitive assessment undertaken by us. The games were developed by us, and through these field trials, we obtained direct insights into the experience and feedback from youths (aged 18-28 years) and older participants (aged >60 years). The choice of a younger group was motivated by evidence of cognitive decline beginning in the third decade of life [58-72]. On the basis of the thematic analysis of the field trial data, we have richer insights into the user perception of VR games for cognitive assessment. We also present and discuss the implications of the findings for the key stakeholders in the field to foster real-world translation of the results, a practical goal that is missing in the aforementioned studies.

## Methods

### Overview

Our thematic analysis was based on the interview data we collected as part of our pilot on VR games among 82 younger participants (aged 18-29 years) and 42 older adult participants (aged ≥60 years). The youths were recruited from BITS Pilani K. K. Birla Goa Campus, and the older adults were recruited from the Annasawmy Mudaliar General Hospital, Bangalore, India.

**Figure 1:**
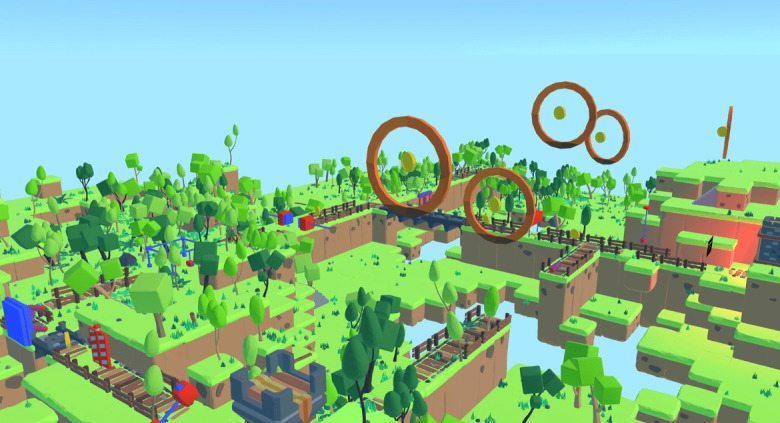
Navigation game sky view. The brown path and course can be seen, and the red and blue game elements indicate obstacles. The orange-colored hoops in the sky with suspended coins inside them illustrate the flying segment of the game.

**Figure 2:**
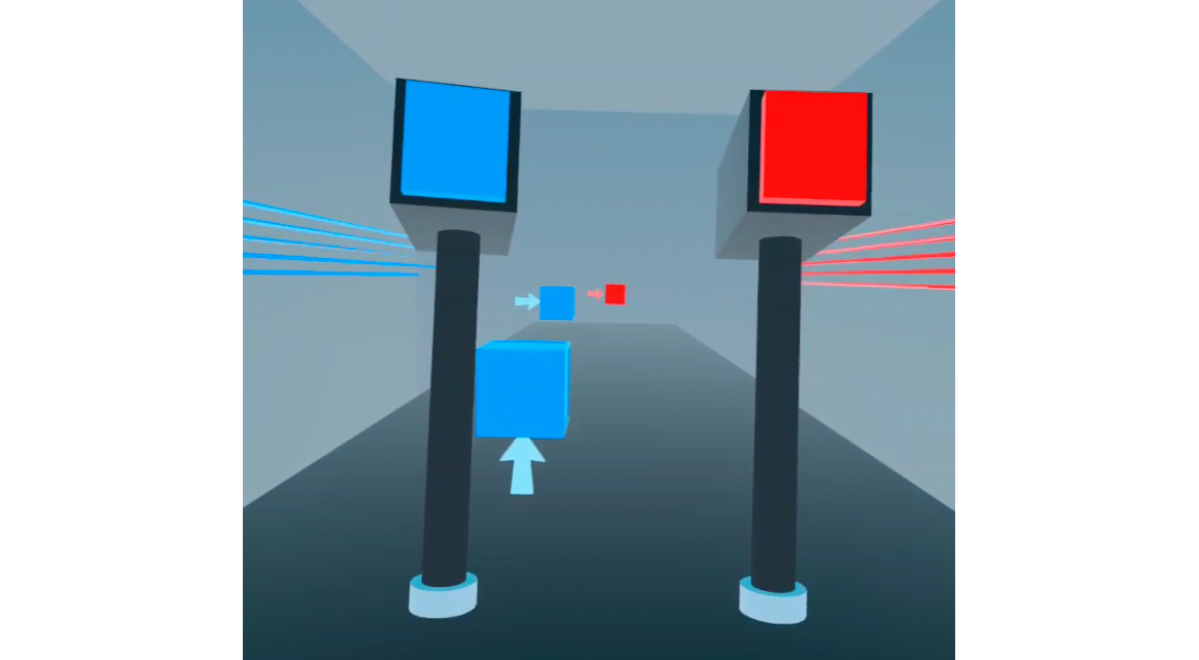
Hand-eye coordination game first-person view. In total, 2 hammers can be seen (red and blue colored); each incoming cube has a direction attached to it that indicates the desired motion of the hammer.

**Figure 3:**
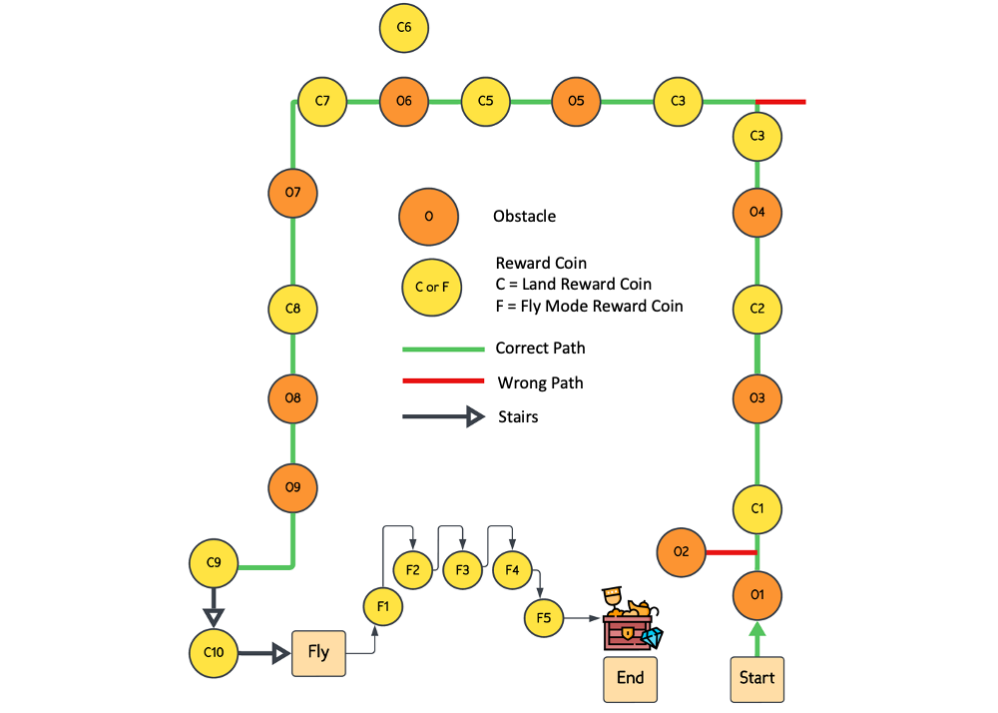
An abstraction of the virtual reality–based navigation game showing the obstacles, rewards (on the land and flying section), correct and incorrect paths, and the flying section.

In total, 2 VR games were piloted: a navigation game (Figure 1) and a hand-eye coordination game (Figure 2). The navigation game is an obstacle course game in which the participant has to wear the VR headset and use the handheld controllers to travel (walk and fly) through an animated virtual world course (land and sky), collect coins (rewards), identify turning points, and avoid obstacles to reach the final treasure (Figure 3). In the flying course, the participant uses controllers to fly and collect the coins in hoops suspended in the air.

In the hand-eye coordination game, the player is expected to hit blue cubes using a blue hammer (VR controller) and red cubes using a red hammer (Figure 2). The blue hammer is in the left hand of the player, and the red one is in the right hand. A correct hit is registered when the red or blue cube is correctly hit by the red or blue hammer respectively in the direction specified on the incoming cube. Any other hit is incorrect. With every correct hit, the speed of the incoming cubes increases. Each participant played 3 trials of each VR game after playing a mini game that served as a tutorial for the actual game. An Oculus VR headset was used.

Both VR games were piloted in the younger group, but only the hand-eye coordination game was piloted in the older group due to the complex gameplay of the navigation game and the preliminary feedback of 2 older adults (aged >60 years). Before game administration, participants’ basic level of proficiency with gaming, VR, and computers was obtained using a 5-point visual analog scale wherein 1 indicated the least experience and 5 indicated maximum experience. These data were important for contextual interpretation of game performance and feedback.

After game administration, feedback was obtained using 3 standard questionnaires: Virtual Reality Sickness Questionnaire [73], Virtual Reality Presence Questionnaire [74], and the System Usability Scale [75]. On the basis of the feedback from these 3 questionnaires, each participant was interviewed to obtain clarity on the context that guided their responses to the questionnaires. These interview data were used for the thematic analysis. The entire process took 1 hour for each participant.

Each individual comment was first coded to summarize its overall idea. Later, codes that were semantically similar were grouped together to form a theme. Themes were identified and later reviewed. Themes were categorized and named to reflect the codes they encompassed. In addition to the thematic analysis, we summarized the scores on the 3 questionnaires for each group. A statistical comparison between the groups was not possible because the older cohort did not play the navigation game. Finally, in a separate section, we discuss the implications of the findings for the key stakeholders in the field.

### Ethical Considerations

Written informed consent to take part in this study was provided by the participants. The procedures contributing to this work comply with the ethical standards of the relevant national and institutional committees on human experimentation and with the Helsinki Declaration of 1975 as revised in 2008. All procedures involving human patients were approved by the Human Ethics Committee of BITS Pilani Goa Campus (reference HEC/BITS Goa/2023-2026). Ethics approval was also obtained from Annasawmy Mudaliar General Hospital separately for the recruitment and assessment of older adults.

## Results

### Overview

A total of 82 younger (mean age 20, SD 2.09 y; median age 20 y; n=67, 82% male) and 42 older (mean age 71, SD 6.31 y; median age 70 y; n=26, 62% female) participants took part in the study. In the younger group, most people (77/82, 94%) were right handed, 4% (3/82) were left handed, and 2% (2/82) were ambidextrous. A total of 96% (79/82) of the participants were pursuing a graduate degree, and 4% (3/82) were pursuing a doctorate. Of the 42 older people, 30 (71%) were married, 8 (19%) were widowed, 3 (7%) were single, and the others’ data could not be found.

The results of the statistical comparison of the gaming, VR, and digital experience of the younger and older cohort are presented in Table 1. For all 3 measures, older people were less experienced than the younger ones. This was an expected observation as young people are relatively more aware of technology and gaming gadgets. However, strangely, the difference was lowest for VR experience. This small difference was due to the unfamiliarity of VR technology in both the younger and older groups. This comparison provides a context to interpret the results of the thematic analysis.

**Figure 4:**
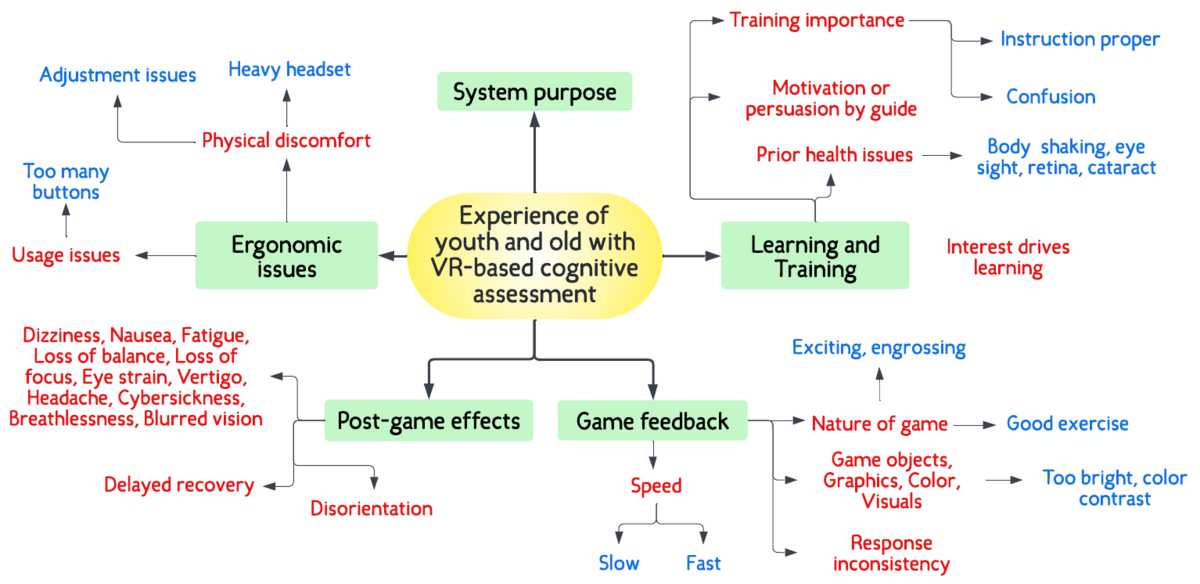
Thematic map showing the 5 themes and their subthemes. VR: virtual reality.

In total, 5 themes (Figure 4) were identified: ergonomic issues, learning and training, postgame effects, game feedback, and system purpose. We discuss each theme and substantiate it using examples from the data.

**Table 1:** Comparison of gaming, digital, and virtual reality (VR) experience of the older and younger cohort. Significance was assumed at *P*<.05.

Measure	*µ*_old_ (n=42)	*µ*_young_ (n=82)	Mean difference (95% CI)	*P* value
Gaming experience	2.85	4.06	–1.20 (–1.68 to –0.72)	<.001
VR experience	1.52	2.22	–0.695 (–1.10 to –0.293)	<.001
Digital experience	3.14	4.59	–1.45 (–1.86 to –1.04)	<.001

### Ergonomic Issues

Most participants reported physical discomfort due to the VR headset or HMD. Older adults who had poor eyesight, retina problems, or cataract surgery history reported greater discomfort and uneasiness. The VR headset is an external device that is worn on the face covering the eyes and has 2 side straps for fixing or tightening the headset with respect to the head. The high weight of this headset (500 g) was the reason for this physical discomfort.

Our observations are substantiated by previous research that has found that the weight of the HMD and high local pressure lead to discomfort and fatigue and upset the user experience [76-79]. Previous research on use of VR HMDs for underground workers suggests that the maximum acceptable mass of the HMD is 1000 g [80], but in our study, we found that participants complained of heaviness in the headset even though its weight was 500 g.

The heaviness of the headset caused discomfort during the hand-eye coordination game in both the younger and older cohorts. We believe that this discomfort was aggravated by the physical movement of hands and limbs required in the hand-eye coordination game because similar discomfort was not reported for the navigation game, which does not require any physical movement. Thus, it appears that games that require passive engagement would be more tolerable with the current weight of the headset. However, such passive engagement is not conducive to realistic cognitive assessment because real-world tasks require movement and action. Therefore, the heaviness of the headset is detrimental to the long-term adoption and acceptance of VR-based cognitive assessment games in the general populace [77,79].

It was also found that the side straps provided with the commercial headset are not user-friendly and using them for fixing and adjusting the headset is inconvenient. The issue of adjustment of straps was reported in the younger cohort, in whom the pilot was conducted first. On the basis of their feedback, we replaced the original adjustment straps with the enhanced side-strap support provided by the KIWI design. This change improved the head adjustment for the older group, and no adjustment problems were reported in this cohort. Our observation on the original adjustment straps provided with the Oculus headset is confirmed by an opinion piece by a law, technology, and human rights attorney [81]. The author mentioned that the strap adjustment piece keeps pulling out when adjusting for different participants and is difficult to fit around traditional headgears such as turbans and hijabs.

In addition to the headset and strap problems, participants reported issues related to button use in the handheld controllers. Each Oculus handheld controller has a total of 6 buttons. The index finger is generally put on the trigger button, the middle finger is put on the gripper, and the thumb is used to control the other 4 buttons. Although the game instructions notify the use of the buttons, the participants still reported confusion with button use at runtime. Particularly in the navigation game, participants reported confusion as the game involved multiple buttons: walking, stopping, and flying. The controller buttons do not seem naturalistic to real-world use, and some researchers indicate that VR gloves are superior [82].

However, for the hand-eye coordination game, which did not require any buttons to be pressed on the controllers, this confusion was not reported, but a peculiarity was observed wherein older people assumed that they needed to press the button to play the game. It took some time to make them realize that only controller movement was required and no buttons on the controller needed to be pressed. However, ideally, in such games, it is better to use controllers that do not have buttons. VR gloves could appear more intuitive and naturalistic as they lessen the abstraction between the real and virtual worlds [82].

Textbox 1 shows some comments from the participants that substantiate the ergonomic issues with the headset.

Due to both the heavy headset and multiple buttons on the controllers, the participants could not seamlessly play the games. To address this, device manufacturers need to minimize the headset weight, and game developers can use the buttons on the controllers that are intuitive and aligned with natural finger use in day-to-day life.

**Comments from the participants on the theme of ergonomic issues.**

**Youths**

- “People with high power in specs, may find some discomfort initially.” [Male and female participants; aged 18-21 years]
- “Headset was heavy.” [Male and female participants; aged 18-26 years]
- “Making turns in the Navigation game using controllers was not very friendly.” [Male and female participants; aged 18-21 years]
- “I had fullness of head due to the headset.” [Male and female participants; aged 19 years]
- “Too many buttons in the controller.” [Male participant, aged 18-19 years, and female participant, aged 26 years]
- “I had headset adjustment issue, due to which I had blurred vision. With spectacles, it was an added difficulty.” [Male and female participants; aged 22, 23, and 26 years]

**Older people**

- “I had a feeling of interference due to glasses. Without glasses, it was difficult as the headset was very heavy.” [Male participant; aged 62 years]
- “Difficult for everyone, would like to use only the computer frequently, but not the VR.” [Female participant; aged 64 years]
- “Could not play the VR Game as experienced discomfort on putting the headset. I also have retina issues so I do not want to play the game.” [Male participant; aged 69 years]
- “First time holding it, so I am very conscious and find it difficult to use.” [Female participant; aged 71 years]
- “I found the headset very heavy, and without glasses also I found it difficult.” [Female participant; aged 75 years]

### Learning and Training

The second theme related to the learning and training required to play the VR-based games. Given that the VR-based experience was novel for both the younger and older groups (VR experience=1.52/5 for the older cohort and 2.22/5 for the younger cohort), learning and training is crucial. A difficult learning process and poor training might dissuade users from adopting the technology.

In our study, training was provided using a short tutorial for the VR games and controller use. Each participant had a chance to play a mini game before playing the 3 trials for each game. The mini game served as a hands-on training for the games. Both younger and older participants acknowledged that the training helped them play the games independently. Few complained of not understanding the video instructions due to audio and background noise. In these cases, extra explanations were provided, but it is essential to eliminate audio and noise from the training and learning videos. Due to playing 3 trials, the participants were able to gradually learn how to play the games.

We also realized that, in the context of VR, training must not be limited to technology-based training. For older people, training can be augmented by explanation, support, and persuasion from the game administrator. These game administrators could be neuropsychologists, neurophysiotherapists, or neuropsychiatrists. For example, in our study, a male participant aged 73 years could not understand how to play the hand-eye coordination game despite repeated instructions. His recent cataract surgery caused him difficulty to apply the instructions to the game. However, with the patience and support of the game administrator, combined with his interest, he was able to successfully learn how to play the game independently.

However, sometimes, repeated assistance may not work if the patient has cognitive impairment. For example, in our study, a male participant aged 62 years with mild cognitive impairment kept forgetting the instructions during the game, so it was challenging to sustain learning due to his medical condition, impeding his gameplay. Therefore, according to the context, the situation, and the interest of the participant, learning and training can be customized for unique cases.

In the existing literature, very few studies have focused on the learning or training required to play VR games. We found research on using VR games for learning and teaching in academic settings [83,84], but research on the difficulty or ease of learning to operate VR and play games on it is missing. Existing studies on VR focus on its usability [85], perception [86], and adoption [87] but fail to comment on the learning and training required to use it. The learning trajectory of VR is important to study in different population groups at whom the products are targeted, especially the older adult group.

Textbox 2 shows comments from the participants to substantiate the aforementioned discussion.

From the comments in Textbox 2, it is clear that youths reported fewer challenges with learning and training for the VR games and the older group faced difficulties. This difference can be attributed to aging effects on learning [88]. Accordingly, solutions and strategies need to be developed to cater to the unique learning needs of the older population.

**Comments from the participants on the theme of learning and training.**

**Youths**

- “Tutorial was enough, games were easy.” [Male participant; aged 18 years]
- “Technical for first-timers.” [Male participant; aged 19-20 years]

**Older group**

- “Something out of routine, so found it difficult.” [Female participant; aged 65 years]
- “The introduction video voice is not clear.” [Female participant; aged 67 years]
- “The instructions in the video were fast.” [Male participant; aged 73 years]
- “The confusion was there for the first two times, later it became clear.” [Male participant; aged 73 years]
- “Was confused in the beginning, as it had been a long time.” [Female participant; aged 65 years]
- “Except for few things in the beginning, it was easy to learn.” [Female participant; aged 69 years]
- “If the person is interested, they will learn quickly.” [Male participant; aged 73 years]
- “Overall, the games were extraordinary. The guide who conducted the test was in a position to articulate well for my understanding and performance.” [Male participant; aged 72 years]

### Postgame Effects

Both the younger and older cohorts reported immediate but subsiding health-related effects after the VR games. This included dizziness, nausea, fatigue, eyestrain, vertigo or headache, cybersickness, breathlessness, blurred vision, loss of balance, loss of focus, and disorientation. Participants with previous health issues such as screen strain reported relatively higher eyestrain after the VR game.

Due to these health effects, participants took some time to become comfortable after the games, normally 30 to 60 seconds, as we observed. The postgame discomforting effects were more commonly reported after the navigation game. These effects could be attributed to the nature of the navigation game, which requires no physical movement. Thus, the player has a perception of forward motion or flying (in the flying segment) in the game while they are actually standing or sitting in the real world. Due to this sensory discrepancy, there is a sense of inertia during the game and disorientation after the game. This sense of inertia seemed to have compounded over the 3 continuous trials of the navigation game, resulting in postgame disorientation and negative health-related effects.

For the hand-eye coordination game, postgame health effects were minimal in the younger and older cohorts. Unlike the navigation game, wherein a sensory discrepancy is present between the player in the game and the one in the real world, in the hand-eye coordination game, this is not so. The movements of hand and limbs expected in the hand-eye coordination game are aligned with the movement in the real world; consequently, there is no sense of disconnection experienced by the player during the game. Therefore, postgame effects such as dizziness, nausea, and disorientation were not observed for the hand-eye coordination game. Still, tiredness and fatigue among the older group were reported, which were indicative of their physical movement during the 3 trials of the game and may not necessarily be negative.

A core reason for the postgame effects of the VR games could be the continuous administration of 3 trials. Continuous VR exposure, especially to the graphic-rich and stimulating environment of the navigation game, can intensify the negative postgame effects [89,90]. Adverse health effects after VR exposure are well documented in research. Our findings are confirmed by previous research that reports cybersickness characterized by visual fatigue, headache, disorientation, dizziness, nausea, and tiredness [91-97]. In scientific terms, these are referred to as VR-induced symptoms and effects [98]. For a detailed study of VR-induced symptoms and effects, readers can refer to the narrative review by Souchet et al [99].

In Textbox 3, we substantiate the postgame effects using comments from the groups. Among the youth, all the comments on postgame effects were related to the navigation game.

Comments from the older cohort are shown in Textbox 4. We noticed very few complaints of postgame adverse health effects among the older group. This is attributed to the gameplay of the hand-eye coordination game, in which no sense of disconnection or discrepancy was experienced by the player in the virtual world and the real world.

From the aforementioned discussion and examples, it is clear that sensory discrepancy or too much stimulation and continuous exposure can lead to negative postgame effects, which normally subside after some time and are not dangerous. Still, these effects may be detrimental to the acceptance of VR games and need to be minimized.

**Comments from the younger participants on the theme of postgame effects.**

**Youths**

- “Eye strain due to color in the Navigation game.” [Male and female participants; aged 18-23 years]
- “Eye strain due to headset.” [Male and female participants; aged 19-23 years]
- “Skeptic of the vision problems caused by VR.” [Male participant; aged 19 years]
- “Exhausted after the Navigation game.” [Male participant; aged 19 years]
- “Experience just like post exam—got tired.” [Male participant; aged 19 years]
- “Lost balance many times in the Navigation game.” [Male and female participants; aged 19-21 years]
- “Sense of balance is lost while in the game, headset is heavy. Reported nausea, disorientation. In the second trial removed the headset once, 3rd trial not played. Asked to be seated and play, but reported discomfort and quit.” [Male participant; aged 21 years]
- “Navigation game was disorienting a bit.” [Male participant; aged 19 years]
- “Headache, eye strain, blurry vision due to heavy usage (by the end of Trial III).” [Male and female participants; aged 18-21 years]
- “Headache during post-game form filling.” [Male participant; aged 19-20 years]
- “Initially in the beginning headache was there, improved with time.” [Male participant; aged 18 years]
- “Little dizziness in the beginning.” [Male and female participants; aged 18-19 years]
- “Dizziness is a put-off.” [Male and female participants; aged 18-21 years]
- “Head feels more heavy after the movement game and during turning motions.” [Male and female participants; aged 18-21 years]
- “Head spinning during the fly.” [Male participant; aged 19 years]
- “Cannot play for longer time.” [Male participant; aged 20 years]
- “After the game, took time to come back to the real world.” [Male participant; aged 18 years]
- “Takes time to get normal.” [Male participant; aged 19 years]
- “After the games, difficulty in walking in the real world. Could not walk in a straight line after the game.” [Male participant; aged 19 years]

**Comments from the older participants on the theme of postgame effects.**

**Older group**

- “Eye strain after playing and headache.” [Female participant; aged 64 years]
- “Played only two levels of the VR game, got very tried after two trials, so wanted to stop.” [Male participant; aged 67 years]
- “Could not play the VR game as she felt dizzy.” [Female participant; aged 75 years]
- “I felt very tired and breathless and wanted to stop after the 1st level but was persuaded to try, but again I got breathless after the 2nd level.” [Female participant; aged 75 years]

### Game Feedback

The game feedback differed for the 2 VR games. Most of the younger participants highlighted that the graphics in the navigation game were too bright and had high contrast. Previous research on VR games has also identified color and contrast as important factors for VR games and linked them to cybersickness [76,94]. Poor graphics such as frequent color change and highly dynamic videos are linked to visual fatigue in VR games [100,101].

When inquired about the realism of the game, participants remarked that the game objects were animated and seemed unreal. We realized that the younger participants defined game realism strictly based on its overlap with the real-world environment and objects. Although this expectation is not unjustified, we believe that it imposes a very rigid definition of realism in the context of VR. On reflection, we realized that realism in the context of VR is a broader concept and may include real-world scenarios, but this criterion is not necessary to establish the realism of VR games. Animated game environments that mimic the nature and intensity of cognitive load observed in the real world also hold realism. Accordingly, even if the objects in the animated game environment do not duplicate real-world objects, if they can assess cognitive processes and skills similarly to real-world cognitive engagement, such animated game environment has realism. From this broader standpoint on realism, we believe that the navigation game held realism as it involved cognitive processes linked to walking, avoiding obstacles, waiting, climbing stairs, and grasping.

Realism in VR has been found to be associated with greater presence and more intense responses [102,103] and is one of the factors that determine enjoyment. Thus, it is important to consider it when designing games for cognitive purposes.

Textbox 5 presents some comments from the younger group on game feedback.

For the hand-eye coordination game, color or graphics issues were not reported by any of the group participants. In fact, a female participant aged 69 years shared that she was deeply involved in the visuals of the hand-eye coordination game. However, participants pointed out a mismatch and inconsistency between user action and game responses. Both younger and older participants complained that their hammer hits were not registered at times, due to which they lost key points. Participants also reported latency in the response to their actions, which caused lagging in the game. Both latency and lagging are known factors that relate to VR experience; however, it is believed that these are hardware issues and are less likely to occur with new HMDs [76].

Furthermore, in the older group, we found mixed reviews on the speed of the incoming cubes in the hand-eye coordination game. Some participants felt that the speed was higher, due to which they could not focus on both the colored cubes, whereas a few felt that it was too slow and could be increased. These 2 contrasting views indicate subjectivity in the perception of the game but also call for dynamic adjustment of the speed of incoming cubes. Such enhancement could greatly improve the user experience of the game. Moreover, given the frailty in the older group, we also noticed that the hand-eye coordination game could be made more flexible by introducing a seated gaming arrangement.

Finally, our broader definition of realism was also confirmed in the hand-eye coordination game, wherein participants reported that the game felt natural and real. Even though no one plays such a game in the real world, the movements, such as lifting a hammer and moving it sideways, up, and down, are typical of the real world. A sense of sensory synergy (ie, similar movements in the real and virtual world during gameplay) also contributed to a sense of realism in the hand-eye coordination game. Our views on realism in the VR games are confirmed in a research paper that calls for deeper understanding of realism and mentions that it relates to both the illusion and immersion components [104]. Thus, a multisensory environment could be more immersive as it engages the senses of the person just like the real world.

Textbox 6 presents some of the comments on the hand-eye coordination game from the older cohort that substantiate this theme.

**Comments from the younger participants on the theme of game feedback.**

**Youths**

- “HD Graphics could improve the user experience.” [Male participant; aged 18 years]
- “Graphics did not seem natural.” [Male participant; aged 18 years]
- “Cartoonish visual aspects, knew they were fake.” [Male participant, aged 19 years, and female participant, aged 21 years]
- “Colors were too bright, too many things were moving.” [Male and female participants; aged 18-19 years]
- “Lot of stimulus was there.” [Male participant; aged 19 years]
- “Interaction with the environment was not realistic.” [Male participant; aged 19 years]
- “Photorealism is lacking.” [Male participant; aged 19-21 years]
- “Graphics not that good, color saturation unlike the real world in the Navigation game.” [Male participant; aged 22 years]

**Comments from the older participants on the theme of game feedback.**

**Older cohort comments on the hand-eye coordination game mechanics**

- “Getting annoyed, sometimes, even when hitting correct, it did not smash. The logic of cube hitting was not consistent for all the cubes.” [Female participant; aged 68 years]
- “The speed was so fast it required to be fast, but that was causing confusion.” [Male participant; aged 73 years]
- “The incoming cubes were too fast, and sometimes when it is left and other is coming at the right, it is difficult to move fast.” [Male participant; aged 68 years]
- “When the game is slow, it is ok, but not when it is fast.” [Male participant; aged 78 years]
- “As soon as I hit red, blue came fast, that’s why I lost so many points. Felt like brain was not acting properly when using two hammers.” [Male participant; aged 73 years]
- “Needed practice, left hand not as fast as the right so took time to be fast.” [Female participant; aged 67 years]
- “The cube could move faster, as it is giving time to think, could be challenging if it moved faster.” [Male participant; aged 77 years]
- “Difficult to coordinate with both the hands, one hand is better.” [Female participant; aged 65 years]
- “As level increases, complexity should be high so that it is interesting and keep the user hooked.” [Male participant; aged 74 years]
- “Was very enjoyable, did not feel like it was a test.” [Female participant; aged 67 years]

**Older cohort comments on the hand-eye coordination game in general**

- “Interesting experience, hand fun. No changes required, good as it is.” [Female participant; aged 63 years]
- “The coordination was a good exercise.” [Female participant; aged 63 years]
- “The game was fun and the music in the game was fun.” [Female participant; aged 68 years]
- “Very much liked it. Nothing was distracting.” [Female participant; aged 69 years]
- “It was a good experience.” [Male participant; aged 74 years]
- “Because the game is artificial environment, it will look artificial.” [Female participant; aged 63 years]
- “If there is an option to let you sit and play, then that would be good.” [Female participant; aged 71 years]

### System Purpose

Both younger and older participants were curious about the purpose of the VR games. At the beginning of the trial, we informed the participants about the games, how to play them, and how participant performance would be mapped to their cognitive abilities or performance. This explanation and training were aimed at making them aware of the subsequent game-*cum*-assessment sessions. After the session, participants were more inquisitive about these games having experienced them directly.

Indeed, the purpose or utility of VR games for users is important because they need to decide whether to accept them for their hedonistic or utilitarian purposes [52,53]. In fact, in the initial stages, the excitement and novelty drove their participation, but going forward, the system’s purpose would define the continuous engagement with the games. Therefore, the system’s purpose is a very important theme in the context of VR-based cognitive assessment games. The purpose indicates the perceived usefulness, which eventually influences their adoption as per the technology acceptance model [105].

A meaningful purpose engenders a positive attitude among the users and key stakeholders and determines the long-term adoption of VR games [106,107]. Thus, after the initial excitement and hedonistic pleasure has plateaued, VR-based games will have to prove their efficacy [57], and a clear purpose can accomplish this.

Textbox 7 shows comments to substantiate the theme of system purpose.

In the older group, no one inquired about the system purpose. It appears that, for them, engagement and an opportunity to try something new were more valuable than purpose, and similar findings have been obtained previously [108-111]. This perspective is advantageous to develop more engaging, active games in the VR environment for older people, especially for rehabilitation and skill building. Thus, a trade-off between utilitarian and hedonistic motives was observed in the 2 groups. Key stakeholders must maneuver their strategies to serve these differential motives.

We have discussed in detail the 5 prominent themes that were identified by analyzing the interview responses of the younger and older groups. We understand the importance of linking these findings to real-world actions and decisions; thus, in the next section, we discuss the implications of the results.

**Comments from participants on the theme of system purpose.**

**Youths**

- “I do not know what the system is for as of now. I do not know why I would use it.” [Male participant; aged 19 years]
- “Not sure what the system is aimed at.” [Male participant; aged 18 years]
- “I won’t use it on a daily basis. Once or twice a week.” [Male participant; aged 20 years]

### Implications of the Results

In this section, based on the results of the thematic analysis, we provide precise actionable suggestions relevant to 4 key stakeholders: researchers, game developers, businesses, and neuropsychology and allied practitioners.

#### Researchers

From the results of the thematic analysis, it is evident that all participants were eager to engage with the VR-based games. To translate this initial eagerness into long-term engagement, researchers need to identify gaming concepts, designs, environments, and abstractions that can be used for developing cognitively stimulating games. Given the limitation of ecological validity in the traditional pen-and-paper–based neuropsychological assessments [8-10,112-114] and the comments on game realism by the participants, researchers can design gaming ideas that resemble real-world situations and mimic the cognitive load and decision-making required for real-world tasks. Such an approach could potentially provide more informed assessment of the real-world–relevant cognitive abilities and deficits of the person. To this end, interaction with customers and brainstorming with other researchers in the fields of neuroscience, computer science, customer satisfaction, and human psychology is essential [115,116].

#### Game Developers

Game developers are often excited about the special effects, high-end graphics, and engaging music that make their games unique. However, in the context of cognitive assessment and rehabilitation, simplicity and intention are key factors that game developers must remember. In the navigation game, button use, graphics, and multiple stimuli caused a lot of confusion and adverse postgame effects, due to which the overall experience was less enjoyable. On the other hand, the hand-eye coordination game was simple, intentional, and very intuitive; consequently, both the younger and older cohorts enjoyed the overall experience. Taking a cue from these findings, game developers must prioritize simple and intuitive gameplay for cognitive engagement. This approach would create games that can flow on their own while engaging the player. This sense of flow is important for the players as it is linked to intention to use [117-120]. Bad graphics, bugs, overstimulated environments, and response-feedback inconsistency can lead to loss of flow and discomfort [45,73,121]. Therefore, game elements must be designed to promote a natural flow in the game. Knowledge of such elements and their quality can be obtained through regular testing among the target users.

#### Businesses

Recently, several business enterprises centered on cognitive assessment and rehabilitation have emerged. To truly have an impact on the lives of people, these enterprises need to focus on some key takeaways from this thematic analysis. The most important takeaway is purpose and meaning. While the technology and game thrill may excite the players momentarily, meaning and the long-term impact of the game will lead to sustainable adoption.

Especially among the younger group, game utility determines adoption. However, in the case of the older group, entertainment and enjoyment seem to have an edge over utility. Still, we believe that the 2 do not have to be mutually exclusive. Both utilitarian and hedonistic aspects can be integrated in cognition-assessing games. For example, our hand-eye coordination game was both useful and enjoyable, and its feedback from both groups was very positive.

Second, manufacturing businesses need to rethink the VR headset and controller design [122,123]. The current weight of the VR headset [124,125] and the adjustment straps can cause issues such as fatigue, headache, and discomfort. Therefore, lightweight HMDs and integrated headsets such as the one with KIWI support are better than soft band straps [124]. In addition, controller buttons must be designed to minimize interference and confusion in use; this improvement would lead to more intuitive use of the controllers, promoting flow in the game [118,119]. Thus, the ergonomic issues directly relate to HMD manufacturers and motivate them to make human-centered design decisions [126-128].

#### Neuropsychology and Allied Practitioners

VR-based games for cognitive assessment and rehabilitation are often used in neuropsychological clinics or laboratories. Findings of our thematic analysis are also relevant to practitioners in these settings. The learning and training theme results emphasize using quality instructions for communicating and demonstrating the game rules. In case audio and video instructions do not suffice, facilitators can explain the instructions to the patients. Our results also demonstrate the positive impact of motivation and persuasion on the patients when they have qualms or apprehension about the games. Neuropsychology practitioners must also be mindful of previous health issues of the patients while administering games to them. Thus, an element of compassion and kindness, not amounting to infantilizing or spoon-feeding, is essential while administering the games, especially in the older cohort.

#### Tabulated Summary of the Theme and Their Relevance to Key Stakeholders

The applicability of the theme results to different sectors were tabulated (Textbox 8). It is clear that most of the themes are relevant to all stakeholders. Thus, all 4 key stakeholders must work in synergy to innovatively use VR technology for cognitive assessment and rehabilitation.

**Themes and the stakeholders directly or indirectly linked to them.**

**Theme and applicability sector or stakeholders**

- Ergonomic issues: hardware manufacturers and businesses
- Learning and training: game developers, businesses, researchers, and neuropsychology and allied practitioners
- Postgame effects: game developers, businesses, researchers, and neuropsychology and allied practitioners
- Game feedback: game developers, researchers, and neuropsychology and allied practitioners
- System purpose: game developers, businesses, researchers, and neuropsychology and allied practitioners

## Discussion

### Principal Findings

In our study, we found that most of the problems faced by the participants were due to headset weight, adjustment straps, game graphics, or motion in the game. Several previous reviews on VR games discuss and describe these challenges associated with VR games but fall short of investigating the reasons behind them [47,48,50]. Such a limited approach does not contribute to improved user experience because we do not know which things to mend and which features to continue with. Furthermore, VR technology is touted to disrupt the health care service sector; therefore, it is crucial that direct human feedback is obtained because any other secondary means, such as literature reviews and web-based reviews [45], risk half-truths.

We also found that different users have different reasons to adopt the product. While both the younger and older cohorts attested to the entertainment and excitement part of it (ie, the hedonistic aspect of the game), the younger group also expressed curiosity about its use and purpose. Previous studies exploring the hedonistic [44] versus utilitarian aspects [48] of VR games provide a general description of these but do not comment on who may be more inclined toward the hedonistic or utilitarian aspects. From our study, we observed that older adults may be more attracted to the hedonistic aspect of the game, whereas the youth may be lured by it temporarily and anticipate utility in the long run. We believe that this insight is extremely useful for evidence-based translation of cognition-assessing VR games.

Our findings on issues of bugs, highly saturated graphics, and confusion due to multiple controller buttons are substantiated by a thematic analysis based on web reviews [45]. Concerns about realism, display quality, and game interface found in our work form the most prominent aspects of VR-based research [46]. Overall, our findings are confirmed by existing research on VR games. However, unlike previous studies that merely report the concerns and advantages of VR-based games, we have raised questions that emphasize the importance of uncovering the causal factors behind these concerns and advantages. Ultimately, the knowledge of these causal factors paves the road for improved experiences with VR game–based cognitive assessment.

Especially with a novel technology such as VR, there is a lot of responsibility on all the stakeholders as there is a risk of addiction [129] and adverse effects [130]. Our discussion on the implications of the study for the stakeholders will indirectly help with the improvement of VR games, thereby assisting in enhanced cognitive assessment and rehabilitation. However, it must be remembered that novel technology tools must be handled with a sense of accountability wherein user interests and safety are superior to commercial interests.

### Conclusions

We presented a thematic analysis of the interview responses of 82 younger (aged 18-28 years) and 42 older (aged >60 years) participants after they played VR-based cognitive assessment games. A total of 5 main themes were identified and discussed: ergonomic issues, postgame health effects, game feedback, learning and training, and system purpose. We found that the younger and older groups had different needs and expectations from these games. For long-term engagement, the younger group prized meaning and utility, whereas the older group liked the enjoyment and entertainment aspects. We also found that the heaviness of the headset, cybersickness, and visual fatigue are the most common problems faced in both groups. However, these problems are less painful if the game environment is not hyperstimulating and has warmer color graphics. In addition, games with less conflict between the real-world sensory information and the VR environment movement are more enjoyable than those with sensorimotor conflict.

We also discussed the implications of these themes for 4 key stakeholders in the field: researchers, game developers, businesses, and neuropsychology and allied practitioners. Researchers must identify real-world concepts that can be used to design ecologically valid games that engage the senses and cognitive abilities similarly to the real world. Game developers need to develop games that are simple, intentional, exciting, and able to flow on their own. Business enterprises must focus on giving a purpose and meaning in these cognitive assessment games to ensure long-term use and impact. Manufacturing businesses must address the issues related to the heaviness of the headset, unfriendly side adjustment straps, and multiple controller buttons to simplify the use experience during assessment. Finally, neuropsychology and allied practitioners play the most important role of administering the VR-based games to the patients and, thus, must be willing to explain these games to patients and use persuasion and compassion during the process. Finally, all stakeholders must collaborate together to develop high-impact games for cognitive assessment and remember to cater the solutions to the unique needs of the target population.

In conclusion, our thematic analysis contributes to the acceptance research on VR-based cognitive assessment games because it compares the feedback from the younger and older groups in primary settings. The discussion on the implications of the findings for the stakeholders provides unique perspectives on translating the findings to the real world. The limitation of the study is the lack of follow-up on game use and adaptation. We believe that a longitudinal monitoring of user attitudes and perceptions would provide a stronger understanding of acceptance and adoption of VR games for cognitive assessment. As a future direction, researchers are advised to monitor game use in the long term, preferably 6 months. In addition, we tested these games in only 2 age groups (18-28 and >60 years) and, therefore, recommend also testing and piloting these games in middle-aged groups (30-50 years). These results would provide more clarity on the overall effectiveness of the VR games to capture age-related cognitive decline. Finally, we piloted these games in healthy groups, so it is strongly suggested to obtain feedback from people with mild cognitive impairment, who are at a greater risk of developing dementia.

Finally, we believe that VR is an immensely novel and exciting tool and it is easy to be swayed by the thrill of technology. Therefore, it is important to remind ourselves to use it in a responsible manner such that human safety and benefits are honored over purely commercial and monetary interests.

## Abbreviations

BITS: Birla Institute of Technology and Science
HMD: head-mounted display
VR: virtual reality
